# Hydrophobia Vera, with Report of a Case

**Published:** 1887-06

**Authors:** James I. Tucker

**Affiliations:** 52 Douglas avenue


THE CHICAGO

MEDICAL JOURNAL AND EXAMINER.

VOL. LIV. JUNE, 1887. No. 6.

### REGULAR COMMUNICATIONS.

### HYDROPHOBIA VERA, WITH REPORT OF A CASE.

BY JAMES I. TUCKER, A. M., M.D.

[Read before the Chicago Medical Society, May 16th.]

During my student years, from 1863 to 1867, the prevailing doctrine in this country seemed to be that there was no such disease as hydrophobia vera—*i. e.*: a disease due to the inoculation and subsequent proliferation of germs of a specific animal-poison which resides in the altered saliva of rabid animals, or, to omit the pathological clause and adopt the definition of Reynolds,* a disease due to a specific animal-poison which resides in the saliva of animals affected with it. This doctrine, I believe, is at the present day very generally held in medical circles. Just now, when the experiments of Pasteur are attracting world-wide attention, and giving rise to heated controversy, every ray of light that can be thrown upon this interesting subject is timely and appropriate. That there is a specific disease caused by the saliva of a rabid

* System of Medicine, vol. 1, p. 711.

animal I have no longer a particle of doubt, or the case which I am about to report has not the importance which I attach to it.

A few years ago I was frequently called to attend a Swedish family residing on Waupanseh avenue, now Thirty-seventh street, in this city. I never entered the house without being greeted with brutal courtesy by a snarling cur. The assurance from his master: "The dog is harmless; he barks but never bites. Down, Tiger!" was an incentive to duty, but hardly sufficient to allay my apprehensions. I did not feel so sure that the dog would not bite, and I was glad enough to get out of the house. Finally the family moved away and I heard no more of them for some time afterward.

One morning about 5 o'clock, Dr. Flavius M. Wilder* called at my office and asked me to go and see a patient with him. I got into his buggy and we drove to Langley avenue. On the way the doctor asked me this question: "Is there, in your opinion, such a disease as hydrophobia?" I replied that the question was still unsettled in my mind; that I had never seen a case and had been taught that the disease commonly called hydrophobia, notwithstanding its classical history and high modern authority, was a form of acute mania with the characteristic delusions and hallucinations.

We entered a cottage and saw lying on a bed the Swede whose family I had attended on Thirty-seventh street a year or two before.

He was rational, but his physiognomy denoted extreme anxiety. His eyes were more brilliant than usual; his pulse rapid and jerky; his tongue coated and dry; he expectorated forcibly from time to time a viscid phlegm, and I observed

* Whose permission was obtained before I wrote this paper.

that his neck was badly scratched, and on uncovering his chest that the skin was lacerated in a horrible manner. Dr. Wilder informed me that the patient had done this with his finger nails during the paroxysms. I was curious to know what these paroxysms might be. Accordingly I went to a cupboard, took therefrom a glass and drew some water into it. Into the water I put a few drops of a tincture. We raised our patient up and requested him to take a couple of teaspoonfuls of this medicine. As soon as he attempted to swallow the first teaspoonful, there followed a paroxysm of dysphagia which it would be difficult to describe. It consisted, in brief, of a dysphagic constriction of the pharynx and œsophagus, in the hope of relieving the agony of which he had torn the flesh from his throat and and chest. This paroxysm was only one of a series which the attending physician had been observing, and which had led him to diagnosticate the case as one of *rabies canina.* Shortly after the patient had another paroxysm, which caused him such extreme suffering that he sprang from his bed, leaped from the window into the garden, and, clad only in his night-shirt, rolled in the dust. Upon being calmed down he exclaimed: "I have dog-disease! It can be none other! It is incurable! It means death. I want you, doctors, to give me something to terminate my life speedily, that I may not suffer unnecessarily!" Thereafter, I am told he made attempts to destroy his life by opening the radial arteries with a needle. Paroxysm after paroxysm, increasing in frequency and intensity, marked the further progress of the disease, and on the following morning after I saw him he died, either from exhaustion or asphyxia—which I cannot say, for he was then unattended except by his family. The intervals between the paroxysms were lucid and rational. He told his wife what to do after his death. He told me that he had been bitten six months before by the same "innocent" cur which he had assured me "barked only, but did not bite." That the cur had been immediately thereafter killed. He attached no importance to the bite till two weeks before we saw him, when, upon going to the lake to bathe he experienced a peculiar constriction about the throat and returned. On the following evening he tried the experiment a second time, and again felt the sensation of constriction. Upon returning he became feverish, the constrictions became more and more frequent and severe, and he directed his wife to send for a physician. If we had not been pre-occupied at the time, we would doubtless have paid closer attention to the more remote phenomena of the disease. We would have looked for the peculiar swellings of the tongue known as *lyssi*, for general hyperæsthesia, exaltation of special senses, priapism, etc.; but, as it was, our attention was absorbed by the observation of the gross appearances. Treatment was ineffectual except as a palliative.

To recapitulate: 1. The man was bitten by a dog, which was immediately thereafter killed; not because he was supposed to be mad, but because his master was angry with him. There was, therefore, no suspicion or dread of hydrophobia.

2. The dog was a sullen, snarling cur, which so many foreigners keep about them as a body-guard. It was unwise to kill him, for it was sacrificing the principal witness.

3. The idea of the biting, and the thought of hydrophobia, did not occur to our patient until the symptoms unmistakably manifested themselves.

4. The difficulty of deglutition was caused, first, by the *sight* of water; second, by the *attempt to drink* water; third, the disease fully developed, the spasms occurred independent of either provocation. There was extreme thirst.

5. The paroxysms, consisting principally of constriction of the pharynx and œsophagus and unexplainable from any other standpoint, psychical or neurological, were almost pathognomonic.

6. Between the paroxysms, there were distinct intermissions, during which the mind was lucid and rational.

7. Rabies confirmed, the phenomena were unmistakable to the physicians and justified the grave suspicions of the patient.

8. No treatment mitigated the symptoms or forefended the result.

9. He died on the morning of the fourth day.

So far as we know at present the prognosis is of the worst description. Death is inevitable. I have not received evidence to convince me of the efficacy of Pasteurization.

It is somewhat remarkable that, if the disease is as common as is popularly supposed—a supposition which the Pasteur inoculations would seem to countenance—how few physicians seem to have seen a single case! In an uninterrupted practice of nearly a quarter of a century I have seen only one case. I have witnessed lyssaform mania, and tetaniform dysphagia and a number of cases of pseudo-hydrophobia, to which the secular press is so fond of giving a sensational coloring, but never before have I seen hydrophobia vera; the symptoms of which are, nevertheless, so unique that no one in the daily habit of practical differential diagnostication can possibly mistake them.

52 Douglas avenue.

